# Emotional blunting and cognitive profile in elderly depressed patients in treatment with vortioxetine

**DOI:** 10.1192/j.eurpsy.2021.875

**Published:** 2021-08-13

**Authors:** F. Franza, B. Solomita, A. Colucci, M. Perito, F. Pellegrino, G. Del Buono, G. Tavormina

**Affiliations:** 1 Sir, Psychiatric Rehabilitation Centre Villa dei Pini, Avellino, Italy; 2 Psychiatry, Psychiatric Rehabilitation Centre Villa dei Pini, Avellino, Italy; 3 Neuroscience, Neamente Association, Mercogliano, Italy; 4 Mental Health Department, UOSM Nocera Inferiore, Nocera Inferiore, Italy; 5 Division Psychiatry, University Salerno, Salerno, Italy; 6 Psychiatric Studies Centre, CenStuPsi, Provaglio d’Iseo, Italy

**Keywords:** Depression, EMOTIONAL BLUNTING, Vortioxetine, Pharmacology

## Abstract

**Introduction:**

Antidepressants in older people have experienced their increase in medical prescriptions in recent decades whit comorbidity with other pathologies and drug polytherapies. With the use of antidepressants, can be observed side and unwanted effects (e.g. emotional blunting). Vortioxetine is a new antidepressant agent which promises fewer side effects.

**Objectives:**

To evaluate the clinical efficacy, safety, side effects (e.g emotional blunting) and cognitive profile

**Methods:**

45 elderly patients affected by MDD (DSM-5) were recruited in our observational study. All patients were treated with vortioxetine for 12 months. Physiological and pathological parameters were collected at baseline (T0), after 3 months (T1), 6 months (T2); 12 months (T3). All patients were administered the following scales: GDS; MMSE; QLi; ODQ. The statistical data were processed with EZAnalyze.

**Results:**

33.33% of patients had a score in the “unlikely depression” GDS group. The ANOVA ODQ “Total” results indicate that at least two of the repeated measures differ significantly. Data of the “antidepressant as cause” dimension are interesting [T0 vs T3 (P-Unadjusted .000; P-Bonferroni .000; T-value 5.687. MMSE scores are indicative of one small but not significant difference. Mean QLIndex scores did not show statistically significant changes, but are indicative of positive changes from the baseline score

**Conclusions:**

Vortioxetine resulted in partial reduction of depression. There was a moderate non-statistically significant increase in body weight, glycidic and lipid profiles. Overall data highlight the importance and role that vortioxetine can have in the management of depressive symptoms in elderly subjects. The handling, effectiveness and reduced side effects of the molecule are emphasized.

